# Association Between Spontaneous Preterm Labor and Genitourinary Tract Infections Among Pregnant Women in a Tertiary Care Hospital in South India: A Cross-Sectional Study

**DOI:** 10.7759/cureus.71973

**Published:** 2024-10-20

**Authors:** PSNRS Sirisha, Shruthi Prashanth, Pavithra Arun, Arivarasan Barathi

**Affiliations:** 1 Obstetrics and Gynaecology, Sri Ramachandra Institute of Higher Education and Research, Chennai, IND; 2 Community and Family Medicine, Employee's State Insurance Corporation (ESIC) Medical College and Hospital, Chennai, IND

**Keywords:** adequate antenatal care, escherichia coli, neonatal sepsis, preterm labor and obstetric care, urinary tract infections

## Abstract

Introduction and objectives

Preterm labor, defined by the WHO as the onset of labor between 20 and 37 weeks of gestation, is a major contributor to neonatal morbidity and mortality, with spontaneous preterm birth accounting for two-thirds of these cases. Genitourinary tract infections, particularly urinary tract infections (UTIs), are a significant risk factor for preterm labor, with studies suggesting that 40% of spontaneous preterm births are associated with genital tract infections. This study aims to determine the association between spontaneous preterm labor and genitourinary tract infections in a sample of antenatal women and evaluate its impact on maternal and neonatal outcomes.

Methods

This cross-sectional study included 144 antenatal women with spontaneous preterm labor, threatened preterm labor, or preterm premature rupture of membranes (PPROM), presenting between 28 and 37 weeks of gestation at Sri Ramachandra Medical College. Midstream urine samples and vaginal swabs were collected for culture and sensitivity testing. Additional parameters, such as maternal age, body mass index (BMI), parity, socioeconomic status (SES), and history of preterm births, were recorded. Blood tests were performed to measure total counts and C-reactive protein (CRP) levels. Maternal and neonatal outcomes, including birth weight and neonatal sepsis, were analyzed.

Results

Of the 144 women, 34 (24%) had a positive UTI culture, with *Escherichia coli* being the most common organism isolated (35%). The majority of UTI-positive cases were observed in women aged 25-29 years (13, 38%) and those from lower socioeconomic classes (22, 65%). A significant association was found between positive UTI cases and elevated total white blood cell counts and CRP levels. Neonatal outcomes in UTI-positive cases included low birth weight (13, 38%) and neonatal sepsis (3, 9%), while maternal complications were mostly limited to asymptomatic bacteriuria and acute cystitis.

Conclusion

The study demonstrates a significant association between spontaneous preterm labor and UTIs. Regular screening for genitourinary infections in pregnancy, particularly in high-risk groups such as women from lower socioeconomic backgrounds, could help reduce the incidence of preterm labor and improve neonatal outcomes. Early diagnosis and treatment of UTIs are crucial for preventing complications in both mothers and neonates.

## Introduction

Preterm labor is the onset of labor after 20 weeks and before 37 weeks of gestation [1]. It is one of the leading causes of perinatal morbidity and mortality worldwide, contributing significantly to neonatal deaths. In 2020, global estimates indicated that preterm birth rates varied between 3% and 6%, with the burden disproportionately affecting low- and middle-income countries [2]. Preterm birth complications are the most frequent cause of death among children under the age of five, accounting for approximately one million deaths annually [3]. Preterm labor can be classified into two main categories: spontaneous and iatrogenic. Spontaneous preterm labor occurs due to various factors, including infections, and constitutes about 70% of all preterm deliveries. The remaining 30% are iatrogenic, induced for medical reasons such as preeclampsia, fetal growth restriction, or placental abruption. Of the spontaneous cases, around 60% result from the spontaneous onset of uterine contractions, while the remainder follow preterm premature rupture of membranes (PPROM).

The etiology of spontaneous preterm labor is multifactorial and remains a challenge to fully understand and predict. However, one of the most consistent and well-documented risk factors is the presence of genitourinary tract infections. These infections, if left undiagnosed or untreated, can have serious repercussions on both maternal and fetal health. In particular, infections of the lower urinary tract, such as asymptomatic bacteriuria, cystitis, and pyelonephritis, have been strongly associated with adverse pregnancy outcomes, including preterm labor, low birth weight, and perinatal mortality [4]. A landmark study conducted in England by Lockwood et al. in 1991 reported that approximately 40% of spontaneous preterm births were linked to genital tract infections [5]. These findings highlight the critical role of infections in triggering the inflammatory pathways that lead to preterm labor.

The biological mechanisms linking genitourinary infections to preterm labor involve both local and systemic inflammatory responses. Infections in the genitourinary tract can lead to the activation of inflammatory cytokines, such as interleukin-6 (IL-6) and tumor necrosis factor-alpha (TNF-α), which in turn stimulate the production of prostaglandins [6,7]. Prostaglandins are known to play a key role in cervical ripening and the induction of uterine contractions, ultimately leading to the onset of labor [8,9]. Infections also contribute to the weakening of the fetal membranes, increasing the likelihood of PPROM, another significant risk factor for preterm birth. Pregnant women are more susceptible to urinary tract infections (UTIs) due to the physiological changes that occur during pregnancy. These changes include dilation of the ureters, decreased bladder tone, and urinary stasis, all of which contribute to an increased risk of bacterial colonization in the urinary tract. The most common pathogen involved in UTIs during pregnancy is *Escherichia coli* (*E. coli*) [10], which is responsible for up to 80% of cases. Other pathogens include *Proteus mirabilis*, *Klebsiella pneumoniae*,* Staphylococcus saprophyticus*, and group B *Streptococcus* (GBS) [11].

Screening and timely treatment of genitourinary infections during pregnancy are crucial in reducing the risk of preterm labor and improving neonatal outcomes. Current guidelines recommend routine screening for asymptomatic bacteriuria in the first trimester of pregnancy, as early detection and treatment with appropriate antibiotics can significantly reduce the likelihood of progression to symptomatic infection or pyelonephritis [12]. Despite this, many cases of UTIs in pregnancy remain undiagnosed, particularly in low-resource settings, where access to screening and healthcare services may be limited. The impact of untreated genitourinary infections on neonatal outcomes cannot be understated.

Preterm infants are at an increased risk of a range of complications, including respiratory distress syndrome, intraventricular hemorrhage, necrotizing enterocolitis, and long-term neurodevelopmental disabilities [13]. These adverse outcomes highlight the importance of identifying and managing risk factors, such as genitourinary infections, in the antenatal period. This study aims to investigate the association between spontaneous preterm labor and genitourinary tract infections in a sample of antenatal women. By evaluating the prevalence of urinary and vaginal infections in women presenting with preterm labor, as well as assessing the impact of these infections on both maternal and neonatal outcomes, this study seeks to contribute to the growing body of evidence on the role of infections in preterm birth. The findings of this research may help inform future strategies for the prevention and management of preterm labor, ultimately improving maternal and neonatal health outcomes.

## Materials and methods

This study was designed as a cross-sectional observational study conducted at Sri Ramachandra Medical College, a tertiary care hospital, between January 2023 and December 2023 for a period of one year. The aim was to explore the association between spontaneous preterm labor and genitourinary tract infections among antenatal women. The study focused on determining the prevalence of urinary and vaginal infections in women presenting with preterm labor and evaluating their impact on both maternal and neonatal outcomes. The study population was consecutively selected to maintain consistency and reliability in the results, ensuring that the inclusion and exclusion criteria were strictly followed.

Study population

A total of 144 antenatal women presenting with spontaneous preterm labor, threatened preterm labor, or PPROM were included. The sample size was calculated with a prevalence of UTI in preterm mothers (49.4%) from a previous literature with 20% relative precision, 80% power, and 95% confidence interval [14]. These women were between 28 and 37 completed weeks of gestation, as confirmed through clinical assessment and ultrasound. All participants provided informed consent, and their demographic, clinical, and laboratory data were collected. Each participant's obstetric history, including parity, body mass index (BMI), and socioeconomic status (SES) (as per the Modified BG Prasad Classification), was noted for analysis [15]. The study was conducted after getting ethics approval from the Institutional Research Ethics Committee of Sri Ramachandra Institute of Higher Education and Research (SRIHER) (approval number: CPS-MED/22/AUG/79/22).

Inclusion and exclusion criteria

Participants included antenatal women with singleton or multiple pregnancies who presented with preterm labor between 28 and 37 completed weeks of gestation. Patients with threatened preterm labor or PPROM were also included. Women who presented with conditions that could complicate labor, such as antepartum hemorrhage, eclampsia, intrauterine fetal death, or known genital tract malignancies, were excluded from the study. Additionally, women with chronic medical conditions unrelated to the genitourinary system were not included to minimize confounding factors.

Sample collection

Two types of samples were collected for laboratory testing: urine samples for detecting UTIs and high vaginal swabs (HVS) for identifying vaginal infections. A midstream clean-catch urine sample was collected in sterile containers from each participant. The urine samples were sent to the hospital laboratory, where one sample was used for routine urine examination and the other for urine culture and sensitivity testing. The urine cultures were incubated overnight at 37°C, and bacterial growth was assessed. Colony-forming units (CFU) greater than 10^5^ CFU/ml were considered indicative of UTI. Additionally, HVS were taken using sterile techniques from the posterior vaginal fornix while the patient was in the dorsal lithotomy position. The vaginal wall was retracted using a Cusco's speculum to collect the sample, which was then sent for culture and sensitivity testing. Vaginal cultures that did not show bacterial growth within 24 hours were considered negative.

Blood investigations

To further investigate the presence of infection or inflammation, blood samples were collected from each participant to measure total white blood cell (WBC) counts and C-reactive protein (CRP) levels. Elevated WBC counts were used as an indicator of infection or an inflammatory response. CRP, an acute-phase protein that rises in response to inflammation, was also measured. A CRP value greater than 0.8 was considered positive, indicating the presence of an inflammatory process.

Clinical and obstetric examination

In addition to laboratory testing, a comprehensive general and systemic examination was performed on all participants. Obstetric examinations focused on assessing gestational age, fetal well-being, uterine contractions, and cervical dilatation and effacement. These examinations were used to evaluate the clinical status of the pregnancy and the likelihood of progression to labor. The study recorded whether patients presented with spontaneous preterm labor, threatened preterm labor, or PPROM, and maternal and fetal outcomes were carefully monitored throughout the pregnancy.

Data collection

Data were collected from each participant regarding demographic details (age, BMI, SES), obstetric history, and clinical presentations. The study also recorded the results of urine and vaginal cultures, total WBC counts, and CRP levels. Maternal outcomes, including the presence of asymptomatic bacteriuria, acute cystitis, pyelonephritis, or puerperal sepsis, were documented. Neonatal outcomes, including birth weight, fetal growth restriction, neonatal sepsis, and non-reassuring fetal status based on non-stress testing, were recorded. The mode of delivery, whether spontaneous vaginal delivery, assisted vaginal delivery, emergency cesarean section, or elective cesarean section, was also noted.

Statistical analysis

Data were entered in Excel (Microsoft Corporation, Redmond, Washington, United States) and analyzed using IBM SPSS Statistics for Windows, Version 26.0 (Released 2019; IBM Corp., Armonk, New York, United States). Descriptive statistics such as mean, standard deviation, and percentages were calculated for continuous and categorical variables. The association between various factors such as age, parity, BMI, SES, and UTIs in preterm labor was analyzed using appropriate statistical tests, including the t-test and chi-squared test. The t-test was used to compare the means of continuous variables, such as total WBC counts and CRP levels, between UTI-positive and UTI-negative groups. The chi-squared/Fisher exact test was employed to examine the relationships between categorical variables, such as SES, parity, and mode of delivery, and the presence of genitourinary infections. A p-value of less than 0.05 was considered statistically significant. Neonatal outcomes, including birth weight and neonatal sepsis, were examined in relation to maternal infection status.

## Results

Table 1 shows that out of the 144 participants, 34 women (24%) had a positive UTI, while 110 women (76%) did not have a UTI. Regarding vaginal infections, all 144 participants (100%) had negative HVS results, indicating no vaginal infections among the study participants.

**Table 1:** Prevalence of genitourinary tract infection among the study participants (n=144) UTI: urinary tract infection; HVS: high vaginal swab

	n	Percentage
UTI		
UTI-positive	34	24%
UTI-negative	110	76%
Vaginal infection		
HVS-positive	0	0%
HVS-negative	144	100%

Table 2 highlights the microorganisms isolated from the 34 women with UTI. *E. coli* was the most commonly isolated organism, found in 12 cases (35%). *Acinetobacter johnsonii* was isolated in seven cases (20%), *Streptococcus* species in four cases (12%), and *Enterococcus faecalis* in three cases (9%). Less frequently isolated organisms included *Enterobacter* species in three cases (9%), *Klebsiella pneumoniae* in two cases (6%), *Citrobacter freundii* in two cases (6%), and *Enterococcus avium* in one case (3%).

**Table 2:** Frequency distribution of various microorganisms isolated from study participants with UTI (n=34) UTI: urinary tract infection

Microorganisms isolated	n	Percentage
Escherichia coli	12	35%
Acinetobacter johnsonii	7	20%
*Streptococcus *species	4	12%
Enterococcus faecalis	3	9%
*Enterobacter *species	3	9%
Klebsiella pneumoniae	2	6%
Citrobacter freundii	2	6%
Enterococcus avium	1	3%

Table 3 presents the association of socio-clinical factors with UTI. Among the age groups, UTI was most prevalent in women aged 25-29 years, with 13 out of 34 women (38%) in this category, though the difference was not statistically significant (p=0.21). SES had a significant association with UTI, as 22 out of 34 women (65%) with UTI belonged to class 4 and 10 out of 34 women (29%) were from class 5, compared to only one woman (3%) from class 2 and none from class 1 (p<0.001). Other factors, including BMI, showed no significant association with UTI, though 26 women (76%) with UTI had a normal BMI (p=0.08). Regarding clinical presentation, 19 out of 34 women (56%) with UTI presented with threatened preterm labor, and eight women (23%) had PPROM. Total WBC counts were significantly higher in the UTI-positive group, with a mean of 14,798.8±3,889.7 compared to 13,160.4±3,747.7 in UTI-negative women (p<0.001). Similarly, CRP levels were elevated in UTI-positive women, with a mean of 1.91±0.28 versus 1.55±0.50 in the UTI-negative group (p=0.02).

**Table 3:** Association of socio-clinical factors with genitourinary tract infection among the study participants (n=144) *Modified BG Prasad Scale 2023 #WHO Classification of Obesity Chi-squared/Fisher exact test Unpaired t-test A p-value of less than 0.05 is considered significant

Characteristic	UTI-positive N (%)	UTI-negative N (%)	Test statistic	P-value
Age group				
19-24 years	11 (32%)	29 (26%)	11.2	0.21
25-29 years	13 (38%)	41 (38%)		
30-34 years	8 (24%)	29 (26%)		
35-39 years	2 (6%)	11 (10%)		
Socioeconomic status				
Class 1	0	10 (9%)	31.92	<0.001
Class 2	1 (3%)	49 (45%)		
Class 3	1 (3%)	7 (6%)		
Class 4	22 (65%)	34 (31%)		
Class 5	10 (29%)	10 (9%)		
BMI category				
Underweight (<18)	0	1 (1%)	12.54	0.08
Normal (18.5-24.9)	26 (76%)	72 (65%)		
Overweight (25-29.9)	5 (15%)	24 (22%)		
Class 1 obesity (30-34.9)	3 (9%)	13 (12%)		
Presenting diagnosis				
Threatened preterm labor	19 (56%)	48 (44%)	4.36	0.14
Preterm premature rupture of membranes	8 (23%)	46 (42%)		
Preterm labor	7 (21%)	16 (14%)		
Parity				
Primipara	18 (53%)	56 (51%)	5.79	0.54
Multipara	16 (47%)	54 (49%)		
No. of births				
Singleton	30 (88%)	93 (84%)	2.56	0.73
Multiple	4 (12%)	17 (16%)		
Previous h/o of preterm births				
No	33 (97%)	103 (94%)	0.66	0.46
Yes	1 (3%)	7 (6%)		
Mode of delivery				
Spontaneous vaginal delivery	18 (53%)	58 (52%)	2.62	0.21
Assisted vaginal delivery	2 (6%)	3 (3%)		
Emergency cesarean section	12 (35%)	45 (41%)		
Elective cesarean section	2 (6%)	4 (4%)		
Total counts (mean+SD)	14798.8+3889.7	13160.4+3747.7	-4.06	<0.001
C-reactive protein (mean+SD)	1.91+0.28	1.55+0.50	-2.21	0.02

Table 4 details the maternal and fetal outcomes among the 34 women with UTI. The most common maternal outcome was asymptomatic bacteriuria, observed in 22 women (64.7%), followed by acute cystitis, which was seen in 12 women (35.3%). There were no cases of pyelonephritis or puerperal sepsis reported in this study. Among fetal outcomes, low birth weight (less than 2.5 kg) was noted in 13 neonates (38.2%), while fetal growth retardation was observed in eight neonates (23.5%). Additionally, six neonates (17.6%) had non-reassuring fetal status (based on non-stress testing), and three neonates (8.7%) developed neonatal sepsis. No perinatal mortality was reported in any of the cases.

**Table 4:** Maternal and fetal outcomes among the study participants with UTI (n=34) UTI: urinary tract infection; NST: non-stress test

Outcomes	N (%)
Maternal	
Asymptomatic bacteriuria	22 (64.7)
Acute cystitis	12 (35.3)
Pyelonephritis	0
Puerperal sepsis	0
Fetal	
Low birth weight (<2.5 kg)	13 (38.2)
Fetal growth retardation	8 (23.5)
Non-reassuring NST	6 (17.6)
Neonatal sepsis	3 (8.7)
Perinatal mortality	0

## Discussion

The study aimed to assess the association between spontaneous preterm labor and genitourinary tract infections among pregnant women. The findings revealed a significant relationship between the occurrence of UTIs and preterm labor, with a prevalence rate of 24% for UTIs among women presenting with preterm labor. This aligns with other studies, such as those by Chhabra and Patil and Kant et al., who reported UTI prevalence rates of 28% and 30%, respectively, in similar populations [16,17], and also at the world level at different regions by Ohuma et al. [18]. The prevalence of UTI among preterm labor patients highlights the importance of screening for and managing infections in pregnancy to improve maternal and neonatal outcomes. The most common pathogen identified in UTI-positive women was *E. coli *(35%), followed by *Acinetobacter johnsonii *(20%) and *Streptococcus *species (12%). This finding is consistent with previous research that identifies *E. coli* as the predominant pathogen in UTIs during pregnancy. A study by Glaser and Schaeffer also reported *E. coli *as the primary cause of UTIs in pregnant women [11]. The high prevalence of *E. coli *can be attributed to its presence in the lower intestinal tract and its ability to ascend into the urinary system, particularly in pregnant women due to physiological changes, such as urinary stasis and hormonal influences.

In this study, most UTI-positive women were aged 25-29 years (38%), with a mean age of 27.76 years. This mirrors findings from other studies by Nassaji et al. and Palupi et al., which also reported the highest UTI rates in this age group [19,20]. The increased risk of UTIs in younger women could be related to hormonal changes, higher sexual activity, and anatomical factors that predispose them to infections. Additionally, women in this age range may have more frequent pregnancies, which increases their exposure to infection risk factors, such as gestational urinary tract changes. A significant association was found between UTI occurrence and SES, with 65% of UTI-positive women belonging to class 4 according to the Modified BG Prasad Classification [15]. This is in line with other research, such as the study by Pavani et al., which also observed that low SES increases the risk of UTIs in pregnancy [21]. Poor hygiene, limited access to healthcare, and undernutrition are likely contributors to the higher incidence of UTIs in lower socioeconomic groups. These findings underscore the need for targeted interventions in lower-income populations, such as improved access to antenatal care and hygiene education, to reduce the risk of infections. In terms of BMI, 76% of UTI-positive women had a normal BMI (18.5-24.9). This is consistent with other studies, such as Nassaji et al., which reported that most UTI-positive women had a normal BMI [19]. While this suggests that BMI might not be a significant predictor of UTI in pregnancy, it is essential to continue monitoring BMI as part of overall maternal health assessments.

The study found that primipara women were more likely to have a UTI, with 53% of UTI-positive women being primipara. This is consistent with other studies, such as those by Michael and Wadhwani and Pavani et al., which also found higher UTI rates in first-time mothers [21,22]. This could be due to anatomical changes, immune responses, or the fact that primipara women may be less aware of UTI symptoms, leading to delayed treatment. Multiparous women might also have developed greater physiological adaptations to pregnancy, reducing their susceptibility to infections. Of the UTI-positive women, 56% presented with threatened preterm labor, while 23% had PPROM. This finding is notable, as infections like UTIs are known to trigger inflammatory processes that lead to uterine contractions and membrane rupture, contributing to preterm labor. The high percentage of women presenting with threatened preterm labor further emphasizes the need for early infection screening and management to prevent preterm birth.

The maternal outcomes in this study showed that 64.7% of UTI-positive women had asymptomatic bacteriuria and 35.3% had acute cystitis. This indicates that asymptomatic bacteriuria is common during pregnancy and can progress to more severe infections if left untreated. No cases of pyelonephritis or puerperal sepsis were reported, suggesting effective management of UTIs in the study population. Fetal outcomes were also affected by maternal UTIs. Low birth weight was observed in 38.2% of neonates born to UTI-positive mothers, with 23.5% showing signs of fetal growth retardation. Neonatal sepsis occurred in 8.7% of cases. These findings are consistent with other studies, such as those by Delzell and Lefevre [23], which highlight the association between maternal UTIs and adverse fetal outcomes, including low birth weight and sepsis. Neonatal sepsis, particularly early-onset sepsis, was linked to maternal UTI, further emphasizing the importance of timely infection control to prevent severe neonatal complications.

A significant association was found between elevated total WBC counts, positive CRP, and UTI in preterm labor. Of the UTI-positive women, 82% had elevated WBC counts, and 94% had positive CRP levels, indicating a strong inflammatory response. These markers are known indicators of infection and inflammation, and their elevation in UTI-positive women highlights the role of infection-induced inflammation in triggering preterm labor. Other studies have shown similar associations, where elevated CRP and WBC levels were predictive of infection-related preterm birth.

Recommendations

Based on the findings, several recommendations can be made for clinical practice. Routine screening for UTIs in pregnant women, particularly those presenting with symptoms of preterm labor or PPROM, should be implemented to detect infections early and prevent complications. This is especially important in women from lower socioeconomic backgrounds, who are at higher risk of infection. The use of antibiotics for confirmed UTIs should be standardized, with follow-up cultures to ensure the complete resolution of the infection.

Limitations of the study

This study has several limitations. Firstly, it was conducted in a single tertiary care hospital, which may limit the generalizability of the findings to other populations, particularly in different geographical or socioeconomic settings. A larger, multi-center study would provide more robust results. Secondly, the study relied on urine culture sensitivity to detect UTIs, which, while effective, might not capture all relevant pathogens, particularly if patients had been pre-treated with antibiotics. Lastly, the study's cross-sectional design limits its ability to establish causality, as it only captures data at a single point in time. A longitudinal design might provide more insight into the progression of UTIs and their impact on preterm labor.

## Conclusions

The study concludes that there is a significant association between spontaneous preterm labor and genitourinary tract infections among pregnant women, with a UTI prevalence of 24% in preterm labor cases. The most commonly isolated pathogen was *E. coli*, and UTI-positive women exhibited elevated inflammatory markers such as total WBC count and CRP. These infections were associated with adverse maternal outcomes like asymptomatic bacteriuria and acute cystitis, as well as fetal complications including low birth weight and neonatal sepsis. Early screening and management of genitourinary infections, particularly in high-risk groups such as women from lower socioeconomic backgrounds, are crucial in reducing the incidence of preterm labor and improving both maternal and neonatal outcomes.
